# The correlation between programmed death-ligand 1 expression and driver gene mutations in NSCLC

**DOI:** 10.18632/oncotarget.15627

**Published:** 2017-02-22

**Authors:** Haitao Yang, Huijuan Chen, Shuimei Luo, Lina Li, Sijing Zhou, Ruifen Shen, Heng Lin, Xianhe Xie

**Affiliations:** ^1^ Department of Chemotherapy, The First Affiliated Hospital of Fujian Medical University, Fuzhou, Fujian 350005, China; ^2^ Department of Oncology, Fuzhou Pulmonary Hospital, Fuzhou, Fujian 350008, China

**Keywords:** PD-L1, EGFR, ALK, KRAS, NSCLC

## Abstract

**Objectives:**

This study aimed to evaluate the correlation between positive PD-L1 expression and driver gene mutations in NSCLC and to seek preliminary evidence in favor of the strategy of PD-L1 inhibitors plus targeted agents.

**Results:**

The overall analyses revealed that positive PD-L1 expression had a significant relationship with KRAS status (RR = 1.26; 95% CI, 1.06−1.50, *P* = 0.010), but no correlation with clinical characteristics (gender, smoking status, histological types), driver gene status (EGFR, ALK) and overall survival (OS): male versus female (RR = 1.16; 95% CI, 0.95−1.42; *P* = 0.15), never smoking versus former/current smoking (RR = 0.79; 95% CI, 0.56−1.11; *P* = 0.17), adenocarcinoma versus non-adenocarcinoma (RR = 0.94; 95% CI, 0.63−1.41; *P* = 0.77), EGFR mutation versus EGFR wild type (RR = 0.74; 95% CI, 0.52−1.06; *P* = 0.10), ALK positive versus ALK negative (RR = 1.02; 95% CI, 0.75−1.38; *P* = 0.91), OS of positive PD-L1 expression versus that of negative PD-L1 expression (HR = 1.31, 95% CI, 0.90−1.90; *P* = 0.15), respectively. Noteworthily, subgroup analyses exhibited that in Chinese cohort studies, positive PD-L1 expression was significantly correlated with OS (HR = 1.75, 95% CI, 1.36−2.24, *P* < 0.0001); and in the studies using PD-L1 monoclonal antibodies (McAbs), positive PD-L1 expression was significantly correlated with KRAS mutation (RR = 1.32, 95% CI, 1.06−1.65, *P* = 0.01) and EGFR mutation (RR = 0.51, 95% CI, 0.28−0.93, *P* = 0.03).

**Materials and Methods:**

After thoroughly searching PubMed, EMBASE and Cochrane Library databases, 11 relevant studies incorporating 3128 cases were identified. The pooled data were analyzed via Review manager 5.3 software.

**Conclusions:**

PD-L1 inhibitors probably was a potential promising option to manage advanced NSCLC harboring KRAS mutation.

## INTRODUCTION

Worldwide, lung cancer, including non-small cell lung cancer (NSCLC) and small cell lung cancer (SCLC), is one of the leading causes of cancer-related deaths [1], and its mortality rate ranks top in China [2]. Moreover, NSCLC is a major histological type accounting for 80%–85% [1]. Because of its high mortality, substantial efforts have been made to prolong overall survival (OS). Initially, conventional platinum-based chemotherapy prolonging a median survival of 8 to 10 months was regarded as a standard regimen, but it hit a plateau with low response of only 20 to 35% and intolerable toxicities [3]. Fortunately, over past decades, the finding of some driver genes in NSCLC, such as epidermal growth factor receptor (EGFR), anaplastic lymphoma kinase (ALK) and kirsten rat sarcoma viral oncogene homolog (KRAS), was a major breakthrough in lung cancer field [4]. Accordingly, molecular targeted therapy, characterized by high efficacy and low toxicity directing to driver gene mutations, has yielded favorable effects on advanced NSCLC. Compared with conventional platinum-based chemotherapy in NSCLC harboring EGFR mutation or ALK rearrangement, targeted agents such as erlotinib and crizotinib have obtained a durable response and extended OS [5, 6]. However, for patients with EGFR wild type or drug resistance to TKIs, the efficacy was poor [7].

Recently, immune checkpoints including programmed death-ligand 1 (PD-L1), programmed death 1 (PD-1), have made waves in anticancer immunotherapy. Some studies exhibited that PD-L1 expressed on the surface of tumor cells, including in NSCLC, melanoma, breast cancer [8, 9]. PD-1/PD-L1 inhibitors, such as nivolumab and atezolizumab, achieved a considerable success in dealing with advanced NSCLC via blocking PD-1/PD-L1 [10, 11]. However, due to the advantaged population of PD-L1 inhibitors remained inconclusive, the response rates were discrepant that inspired oncologists to identify the appropriate individuals. Recently, some researchers investigated the correlation between PD-L1 expression and driver gene mutations, but it remained to be clarified [12–22]. Although three previous studies [23–25] had evaluated the correlation between positive PD-L1 expression and OS, no meta-analysis had been performed on the correlation between positive PD-L1 expression and driver gene mutations. On that account, we performed this study to evaluate the correlation, to screen the potential advantaged population of PD-L1 inhibitors and to find preliminary evidence in favor of the strategy of PD-L1 inhibitors plus target agents.

## RESULTS

### Study selection and characteristics

**Figure 1:**
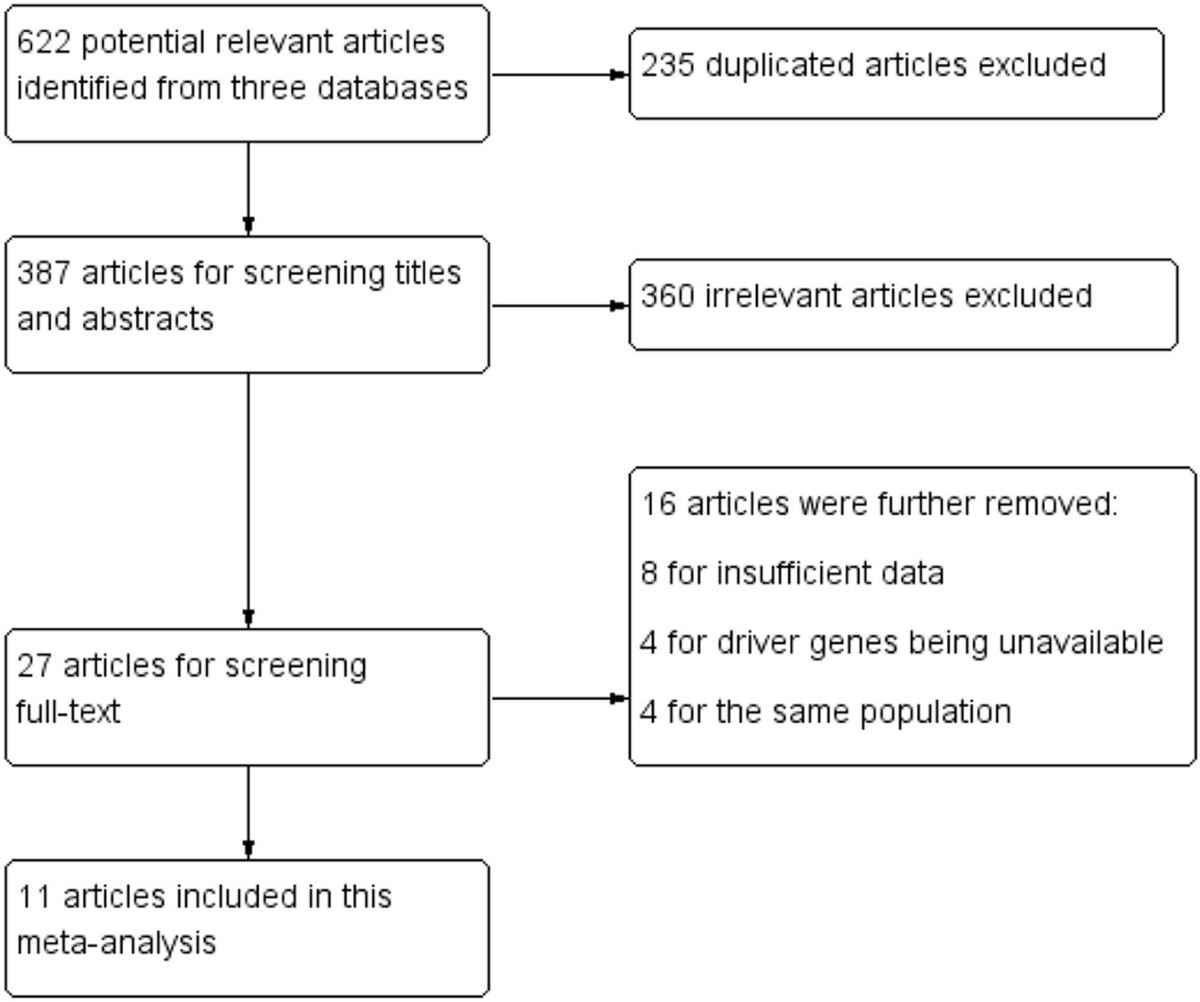
Flow chart for study selection

After initial comprehensively searching from PubMed, EMBASE and Cochrane Library databases, 622 potential relevant articles were found, whereas 595 (235 duplications and 360 irrelevant articles) were excluded. Then, another 16 articles were further removed after screening full-text for the following reasons: 8 for insufficient data, 4 for driver genes unavailable and 4 for the same population. Eventually, 11 articles incorporating 3128 cases were identified. The flow chart of literature searching and the clinical characteristics of included studies were listed in Figure 1 and Table 1, respectively. Of these eligible studies, 5 were from China [17, 18, 20–22], 2 from Japan [16, 19], 2 from Australia [12, 13], 1 from USA [15] and 1 from Italy [14]. The year of publication ranged from 2014 to 2016. The sample size of the included studies ranged from 100 to 678. With regard to the histological type of NSCLC, 7 studies [15–19, 21, 22] evaluated adenocarcinoma (ADC), and the other studies [12–14, 20] involved ADC, squamous cell cancer, large cell cancer and mixed types. Among these studies, positive PD-L1 expression appeared in 28.3% of male, 30.3% of female, 36.0% of never smoking, 32.1% of former/current smoking, 28.9% of ADC and 18.6% of non-ADC. Considering the diversity of PD-L1 antibodies, including 4 studies [14, 17, 21, 22] used polyclonal antibodies (PoAbs) and 7 [12, 13, 15, 16, 18–20] utilized monoclonal antibodies (McAbs), we conducted further subgroup analyses based on antibody types (McAbs or PoAbs). The details of PD-L1 antibodies in each study and the cut-off of PD-L1 were listed in Table 2.

**Table 1:** The characteristics of these included studies

Study ID	Country	Number of samples	Gender	Smoking status	Histology	PD-L1 expression	EGFR status	KRAS status	ALK status	OS
			M/F	Never/(Former/ current)	ADC/ non-ADC	N/P	Wild type /mutation	N/P	N/P	
Cooper et al, 2015 [13]	Australia	678	477/201	NA/NA	276/402	628/50	237/33	182/88	267/3	HR and 95% CI
Ji et al, 2016 [17]	China	100	51/49	74/26	100/0	60/40	40/60	90/10	NA/NA	HR and 95% CI
Yang et al, 2014 [21]	China	163	54/109	132/31	163/0	98/65	66/97	155/8	3/160	survival curves
D'Incecco et al, 2015 [14]	Italy	123	66/57	(never/former)94/current 17	82/41	55/68	67/56	95/28	113/10	survival curves
Song et al, 2016 [18]	China	385	198/187	235/150	385/0	199/186	180/205	369/16	367/18	HR and 95% CI
Zhang et al, 2014 [22]	China	143	59/84	94/49	170/0	73/70	67/76	136/7	134/9	survival curves
Tang et al, 2015 [20]	China	170	93/77	113/57	145/25	58/112	71/99	NA/NA	NA/NA	HR and 95% CI
Inamura et al, 2016 [16]	Japan	268	142/126	112/156	268/0	225/43	97/93	168/21	258/10	HR and 95% CI
Takada et al, 2016[19]	Japan	417	205/212	218/199	417/0	332/85	123/112	NA/NA	NA/NA	HR and 95% CI
Huynh et al, 2016 [15]	USA	261	90/171	NA/NA	261/0	166/95	207/54	153/108	257/4	survival curves
Ameratunga et al, 2016 [12]	Australia	420	297/123	27/376	185/235	320/100	397/23	341/79	NA/NA	survival curves

Abbreviation: M/F: male/female; ADC: adenocarcinoma; non-ADC: non-adenocarcinoma; N/P: negative/positive; NA: unavailable.

**Table 2:** The details of PD-L1 antibodies in each study and the cut-off of PD-L1

Study ID	Method	Types of antibody	PD-L1 antibodies	The cut-off of PD-L1
Cooper et al, 2015	IHC	monoclonal	Mouse monoclonal anti-PD-L1 primary antibody (Merck; clone22C3)	PD-L1 staining intensity ≥ 50%
Ji et al, 2016	IHC	polyclonal	Mouse polyclonal antibodies (Abcam, Cambridge, UK, ab174838)	cases with staining intensity ≥ 2 in more than 5% of tumor cells were considered as positive
Yang et al, 2014	IHC	polyclonal	Rabbit anti-PD-L1 antibody (Proteintech Group Inc. Chicago, IL, USA, 17952-1-AP)	membranous staining was present in ≥ 5% of the cells.
D'Incecco et al, 2015	IHC	polyclonal	Rabbit primary antibodies PD-L1 (CD274) ab58810 (Abcam, Cambridge, UK)	all cases with staining intensity ≥ 2 in more than 5% of tumour cells were considered as positive
Song et al, 2016	IHC	monoclonal	Rabbit anti-PD-L1 primary antibody (Proteintech Group Inc, Chicago, IL, USA, Catalog number: 66248-1-Ig)	positive, when membranous staining was present in ≥ 5 % of the cells.
Zhang et al, 2014	IHC	polyclonal	primary anti-PD-L1 antibody (SAB2900365; Sigma-Aldrich)	positive cut off quickscore of ≥ 3
Tang et al, 2015	IHC	monoclonal	Rabbit monoclonal anti-human antibody (E1L3N™, Cell Signaling Technology, Danvers, MA)	A 5% proportion of membrane-positive tumor cells which were defined as H-score ≥ 5 have been used as cutoff for PD-L1 positivity
Huynh et al, 2016	IHC	monoclonal	Monoclonal antibody (E1L3N, Cell Signaling Technology, Danvers, MA)	Positive PD-L1 expression on tumor cells for 5% cutoff
Inamura et al, 2016	IHC	monoclonal	anti-PD-L1 rabbit monoclonal antibody (clone: E1L3N, Cell Signaling Technology, Danvers, MA, USA)	A score of 5% or more was categorized as PD-L1-positive
Ameratunga M, et al, 2015	IHC	monoclonal	Anti-PD-L1 rabbit IgG (E1L3N) cat # 13684, Cell Signaling Technology	PD-L1 positivity was defined as > 5% cells with membranous staining of intensity 2. Strong positivity was defined as of 50% cells with membranous staining of intensity 2.
Takada et al, 2016	IHC	monoclonal	Rabbit monoclonal antibody (clone SP142, Spring Bioscience, Ventana, Tucson, AZ, USA).	Positive PD-L1 protein expression for 5% cutoff

### Overall and subgroup analyses

#### Correlation between positive PD-L1 expression and clinical characteristics

**Figure 2:**
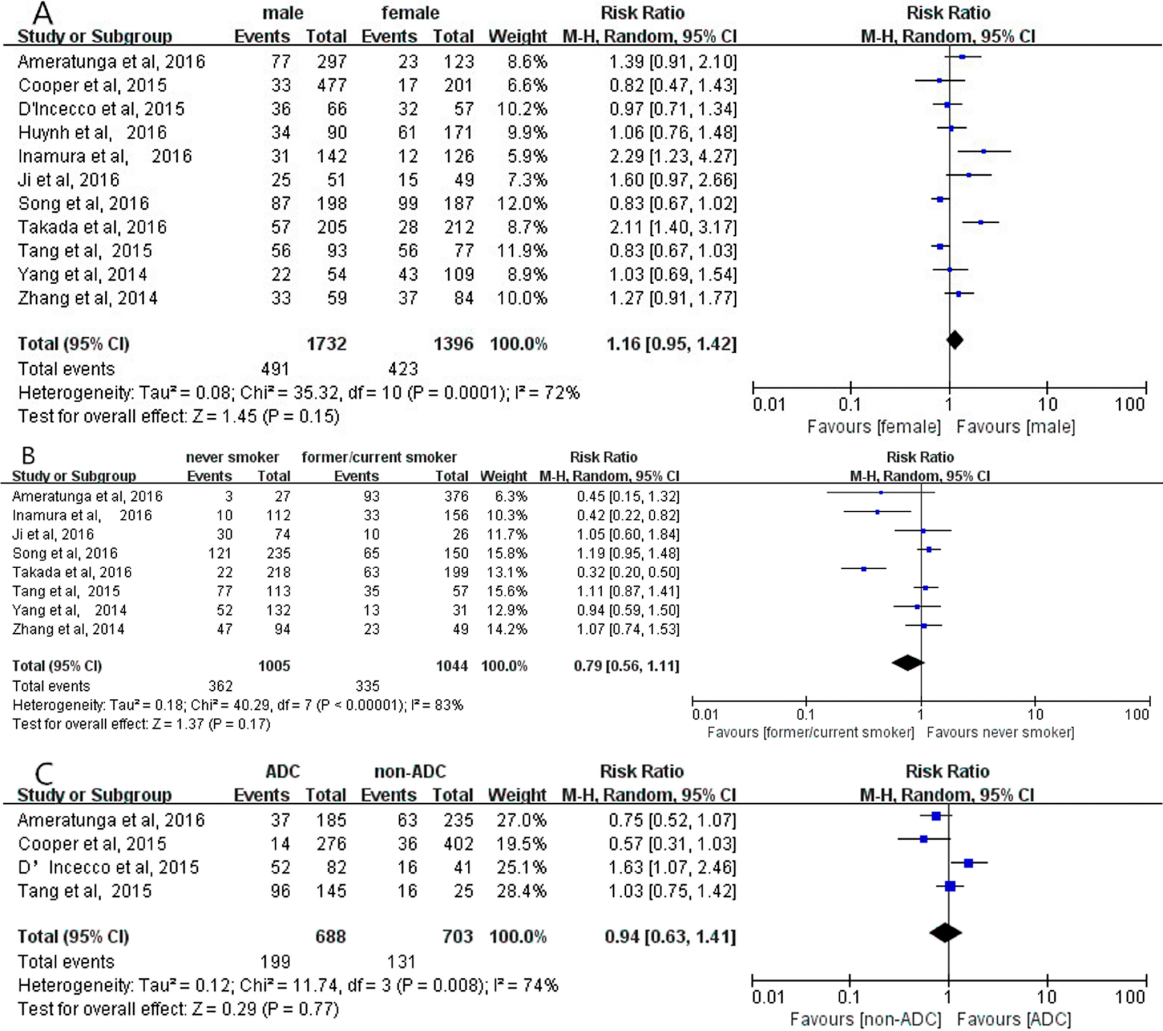
The correlation between positive PD-L1 expression and gender (**A**), smoking status (**B**), histology (**C**) in overall analyses.

**Figure 3:**
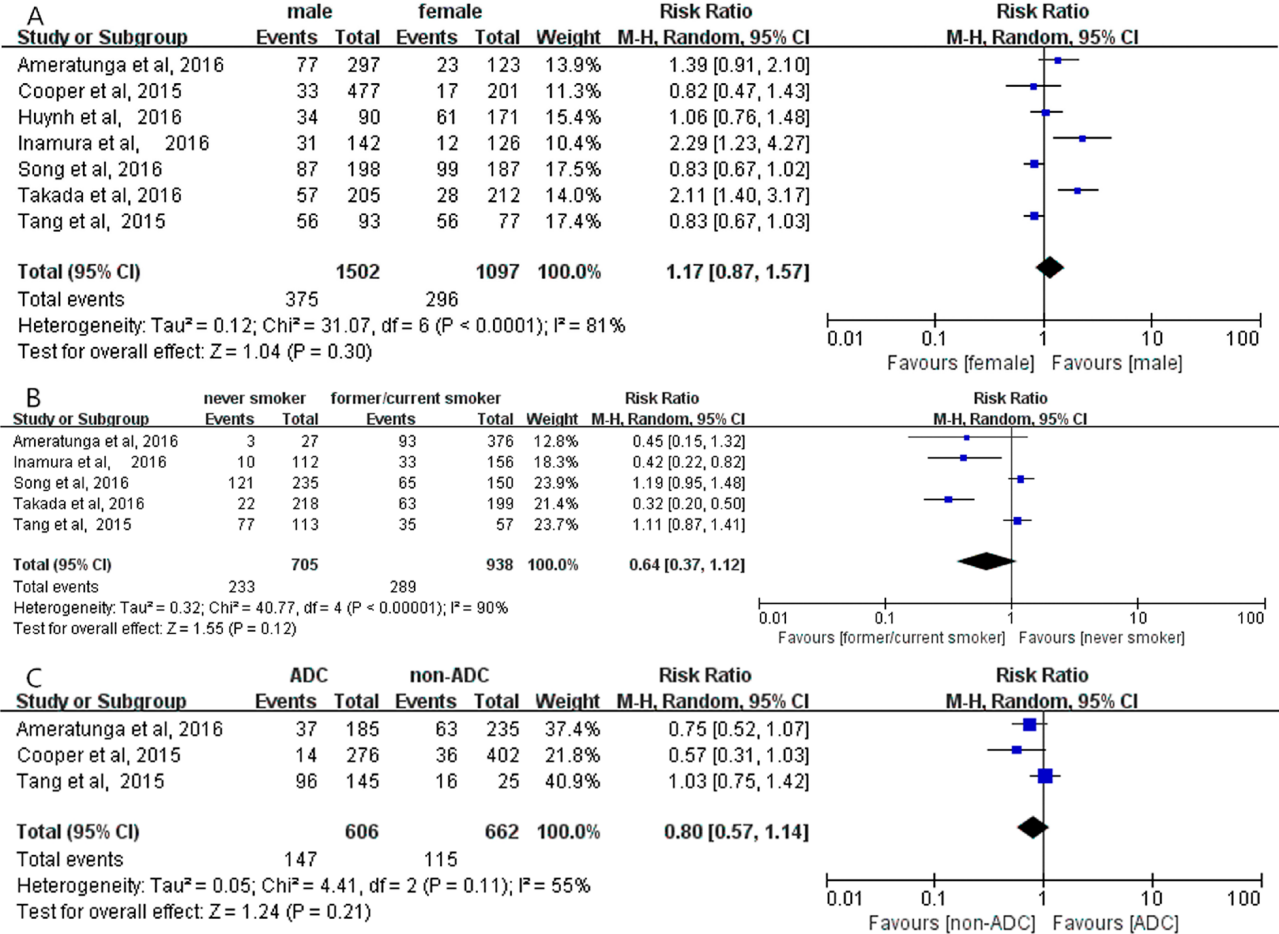
The correlation between positive PD-L1 expression and gender (**A**), smoking status (**B**), histology (**C**) in the studies using PD-L1 McAbs.

**Figure 4:**
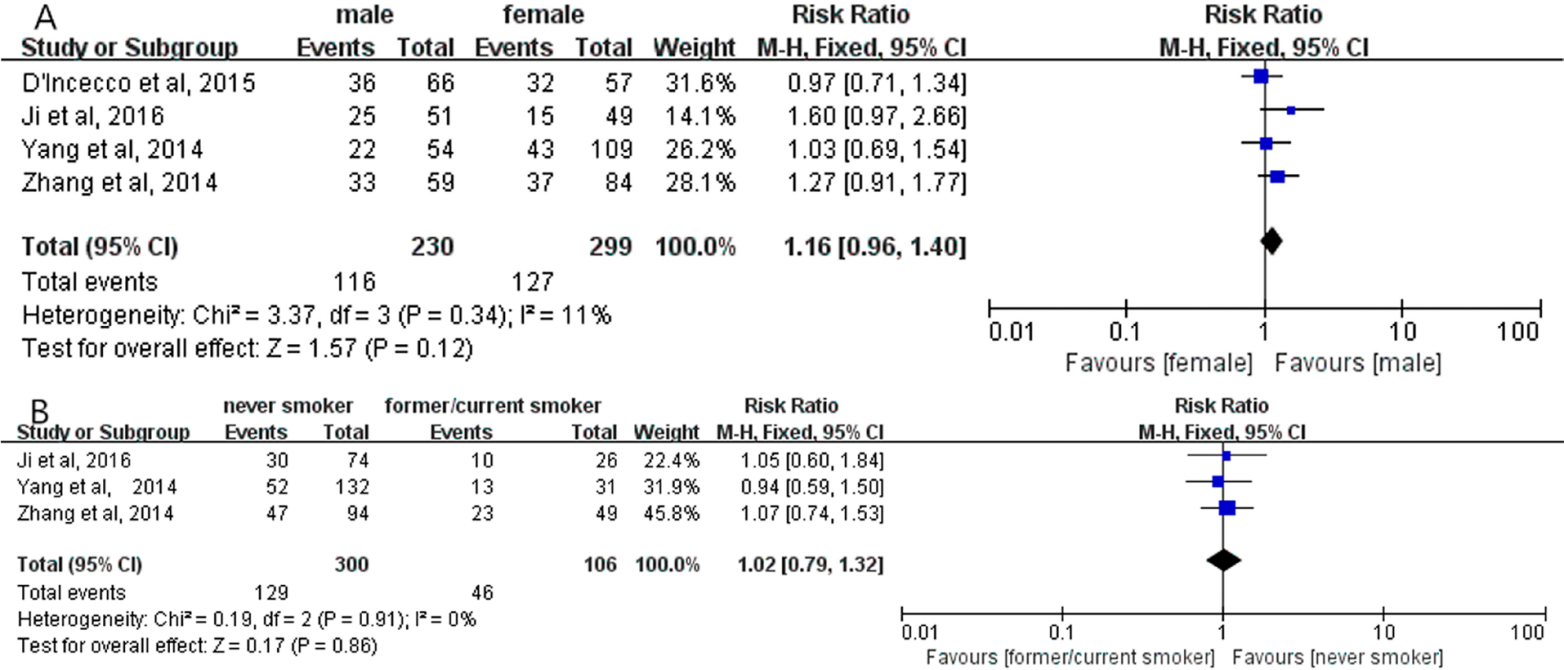
The correlation between positive PD-L1 expression and gender (**A**), smoking status (**B**) in the studies using PD-L1 PoAbs.

**Figure 5:**
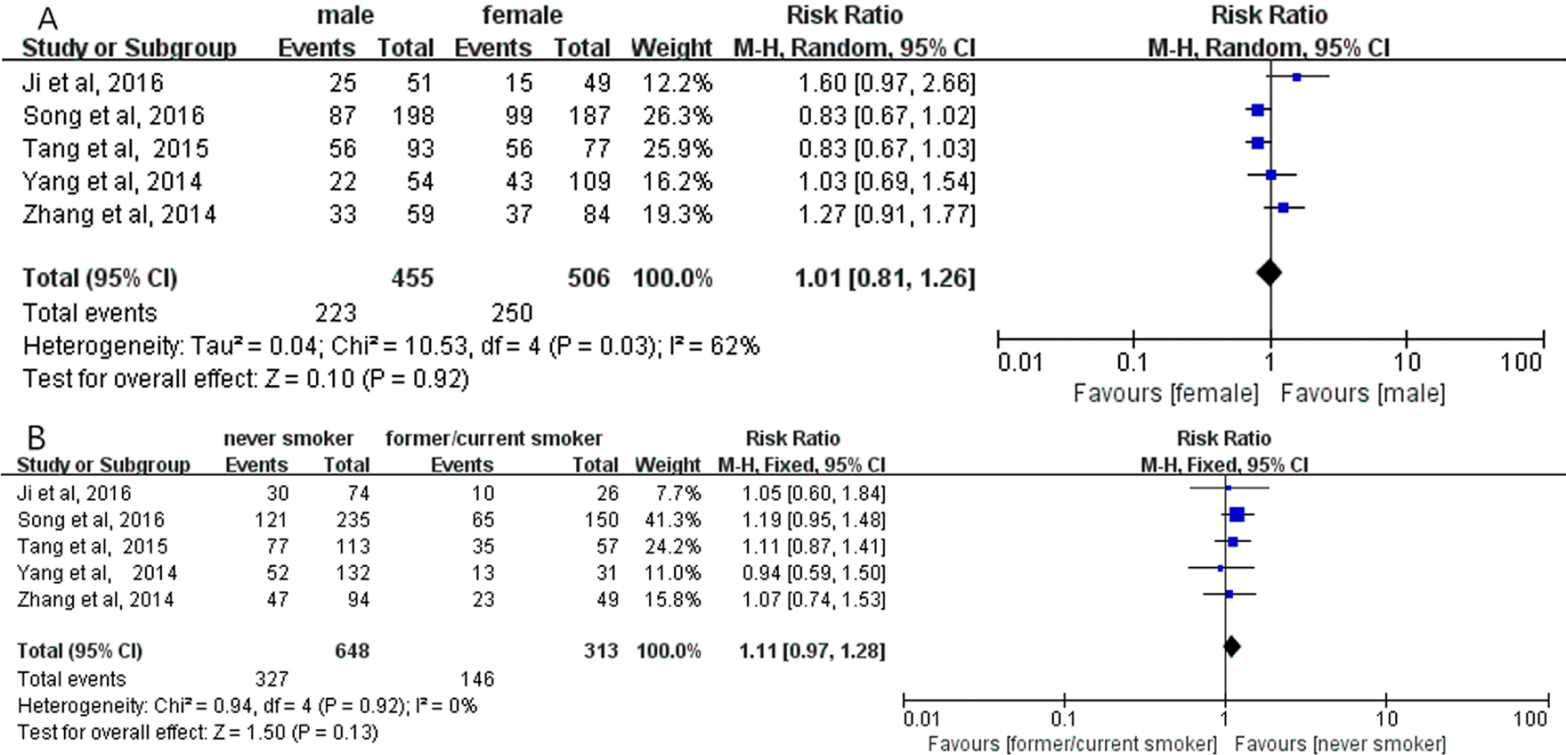
The correlation between positive PD-L1 expression and gender (**A**), smoking status (**B**) in Chinese cohort studies.

Overall analyses revealed no significant correlation between positive PD-L1 expression and gender (RR = 1.16; 95% CI, 0.95–1.42; *P* = 0.15, Figure 2A) [12–22]; smoking status (RR = 0.79; 95% CI, 0.56–1.11; *P* = 0.17, Figure 2B) [12, 16–22]; histological types (RR = 0.94; 95% CI, 0.63–1.41; *P* = 0.77, Figure 2C) [12–14, 20], respectively. Meanwhile, no significant correlation between positive PD-L1 expression and gender, smoking status, histological types were observed in subgroup analyses on the studies using PD-L1 McAbs (Figure 3), on the studies using PD-L1 PoAbs (Figure 4), on Chinese cohort studies (Figure 5).

### Correlation between positive PD-L1 expression and driver genes

#### Positive PD-L1 expression and EGFR status

**Figure 6:**
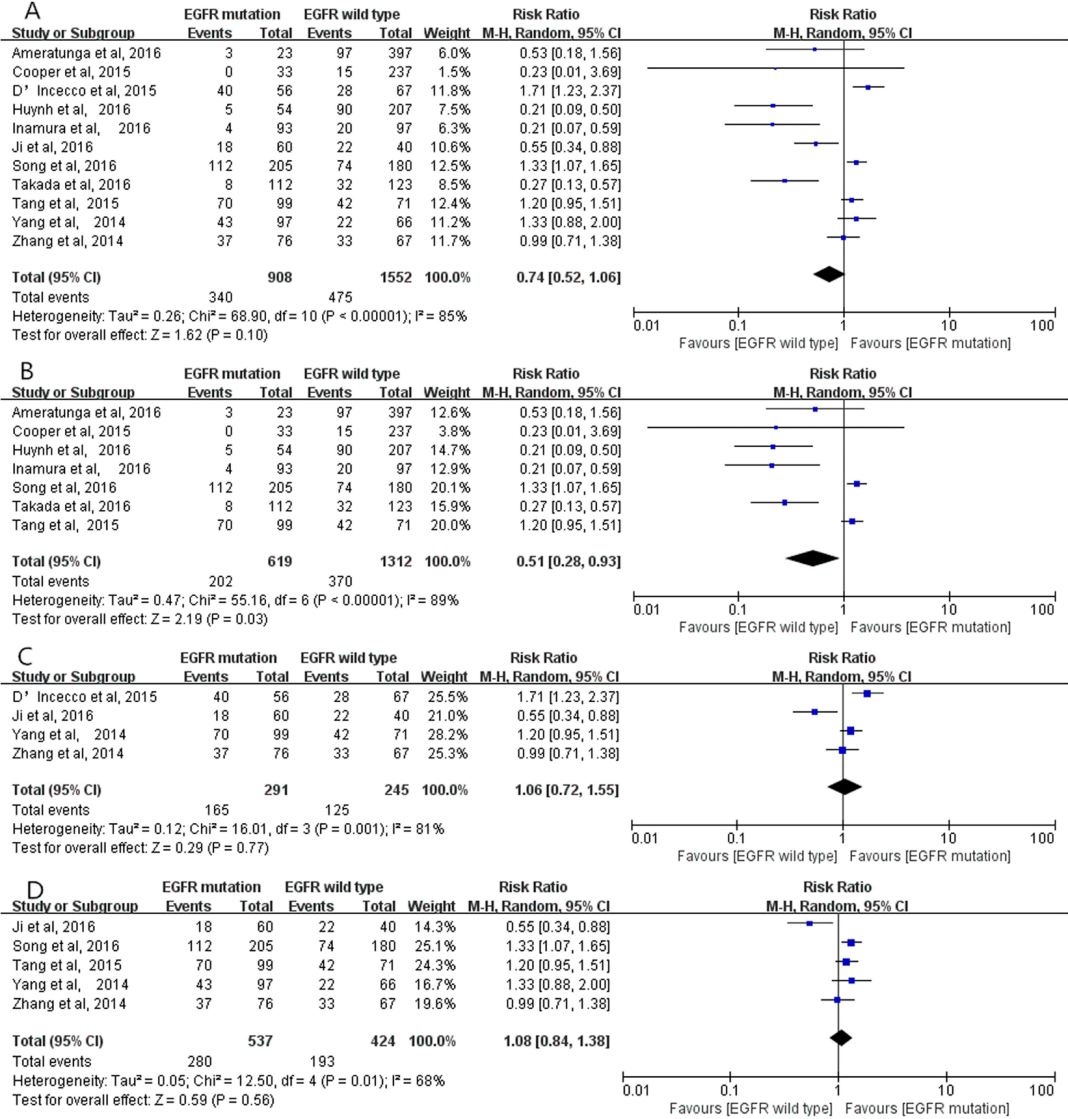
The correlation between positive PD-L1 expression and EGFR status in overall analysis (**A**), in the studies using PD-L1 McAbs (**B**), in the studies using PD-L1 PoAbs (**C**) and in Chinese cohort studies (**D**).

The pooled analysis of 11 studies [12–22] showed no significant relationship between positive PD-L1 expression and EGFR mutation (RR = 0.74; 95% CI, 0.52–1.06; *P* = 0.10, Figure 6A), although positive PD-L1 expression occurred more frequently in EGFR mutation studies than EGFR wild type studies (37.4% versus 30.6%). However, considering the fact that obvious heterogeneity existed among these eligible studies (*I*^2^ = 85%, *P* < 0.00001), subgroup analyses were conducted based on the studies using PD-L1 McAbs or PoAbs, and on Chinese cohort studies. The outcomes demonstrated that in the studies using PD-L1 McAbs, positive PD-L1 expression more frequently occurred in EGFR mutation group than in wild type group (RR = 0.51; 95% CI, 0.28–0.93; *P* = 0.03, Figure 6B) [12, 13, 15, 16, 18–20] while the same results were not observed in subgroup analyses on the studies using PD-L1 PoAbs (RR = 1.06; 95% CI, 0.72–1.55; *P* = 0.77, Figure 6C) [14, 17, 21, 22], and on Chinese cohort studies (RR = 1.08; 95% CI, 0.84–1.38; *P* = 0.56, Figure 6D) [17, 18, 20–22].

### Positive PD-L1 expression and ALK status

**Figure 7:**
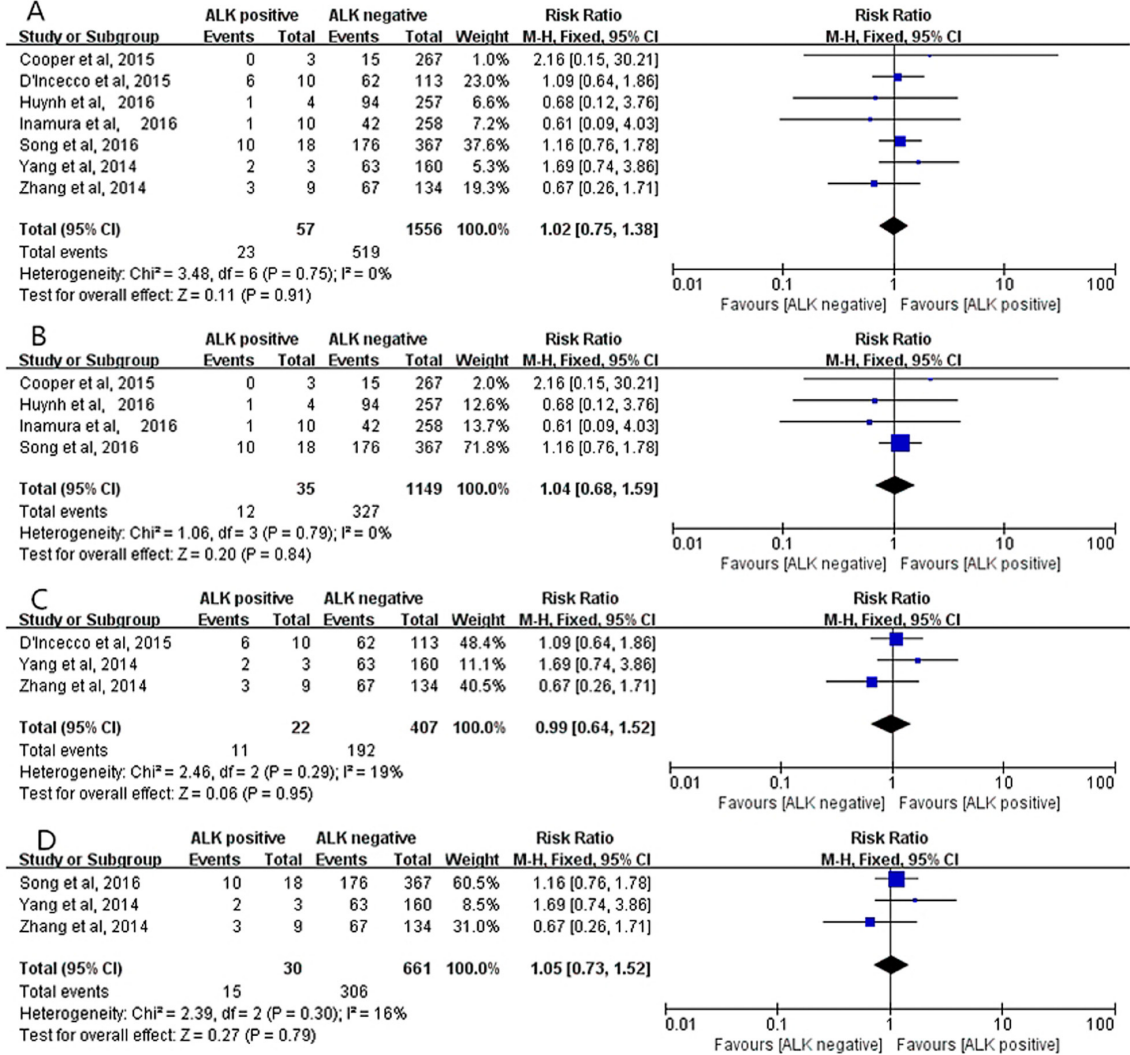
The correlation between positive PD-L1 expression and ALK status in overall analysis (**A**), in the studies using PD-L1 McAbs (**B**), in the studies using PD-L1 PoAbs (**C**) and in Chinese cohort studies (**D**).

Seven studies (1613 cases) [13–16, 18, 21, 22] were applied to assess the correlation between positive PD-L1 expression and ALK status. No heterogeneity existed in these studies (*I*^2^ = 0%, *P* = 0.75), thus a fixed-effect model was employed. The pooled result indicated that positive PD-L1 expression was not associated with ALK status (RR = 1.02; 95% CI, 0.75–1.38; *P* = 0.91, Figure 7A). Moreover, all subgroup analyses suggested that no significant correlation between positive PD-L1 expression and ALK status in the studies using PD-L1 McAbs (RR = 1.04; 95% CI, 0.68–1.59; *P* = 0.84, Figure 7B) [13, 15, 16, 18], in the studies using PD-L1 PoAbs (RR=0.99; 95% CI, 0.64–1.52; *P* = 0.95, Figure 7C) [14, 21, 22], and in Chinese cohort studies (RR = 1.05; 95% CI, 0.73–1.52; *P* = 0.79, Figure 7D) [18, 21, 22].

### Positive PD-L1 expression and KRAS status

**Figure 8:**
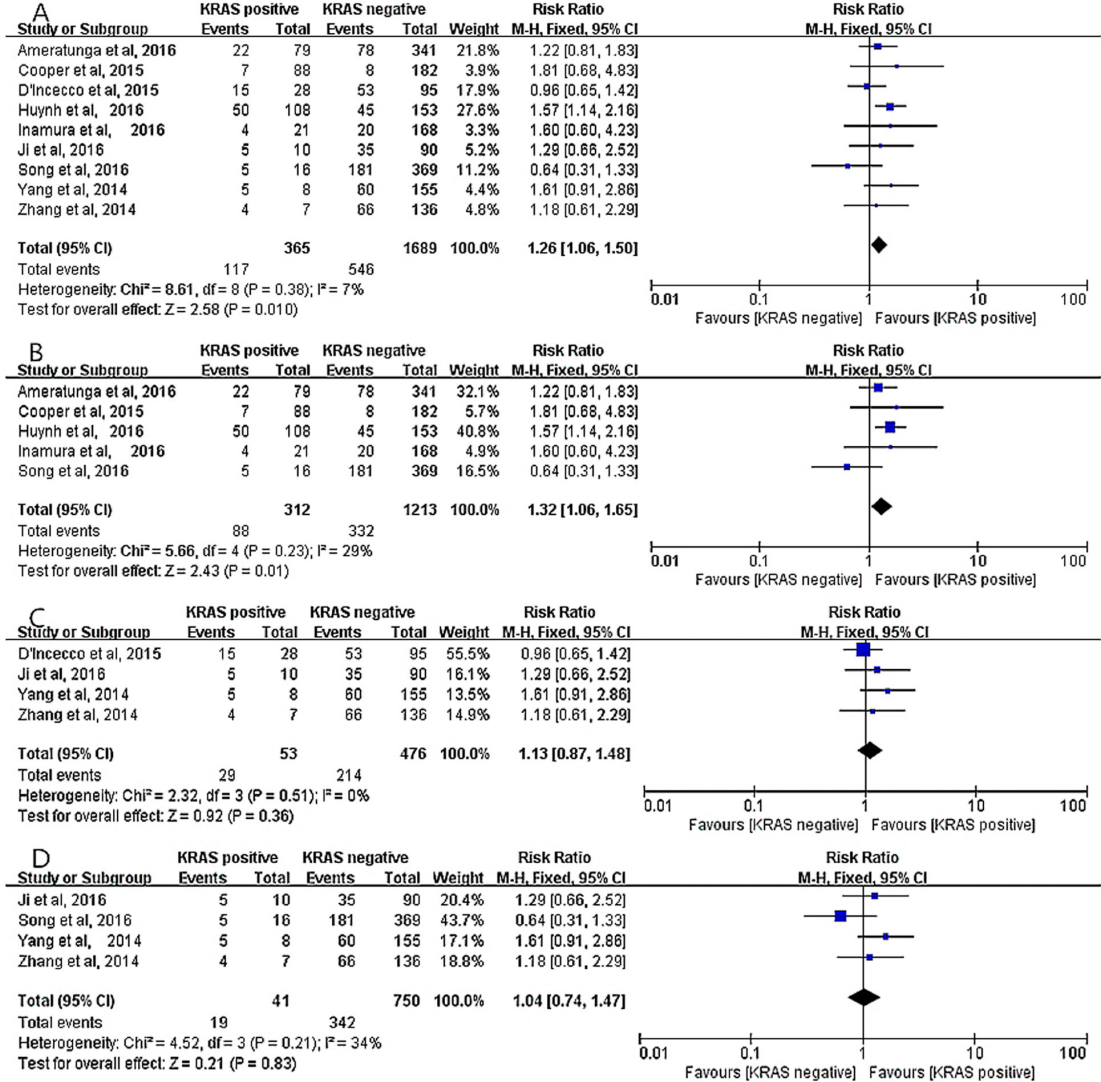
The correlation between positive PD-L1 expression and KRAS status in overall analysis (**A**), in the studies using PD-L1 McAbs (**B**), in the studies using PD-L1 PoAbs (**C**) and in Chinese cohort studies (**D**).

Nine studies [12–18, 21, 22] incorporating 2054 cases were assigned to analyze the relationship between positive PD-L1 expression and KRAS status. A fixed-effect model was employed due to no evident heterogeneity (*I*^2^ = 7%, *P* = 0.38), and the outcome revealed a significant correlation between PD-L1 expression and KRAS status (RR = 1.26; 95% CI, 1.06–1.50, *P* = 0.010, Figure 8A). Furthermore, the result mentioned above was obtained in the studies using PD-L1 McAbs (RR = 1.32; 95% CI, 1.06–1.65, *P* = 0.01, Figure 8B) [12, 13, 15, 16, 18]. However, subgroup analyses on the studies using PD-L1 PoAbs [14, 17, 21, 22] and on Chinese cohort studies [17, 18, 21, 22] displayed that no significant correlation between PD-L1 expression and KRAS status, (RR = 1.13; 95% CI, 0.87–1.48; *P* = 0.36, Figure 8C), (RR = 1.04; 95% CI, 0.74–1.47; *P* = 0.83, Figure 8D), respectively.

### Correlation between positive PD-L1 expression and OS

**Figure 9:**
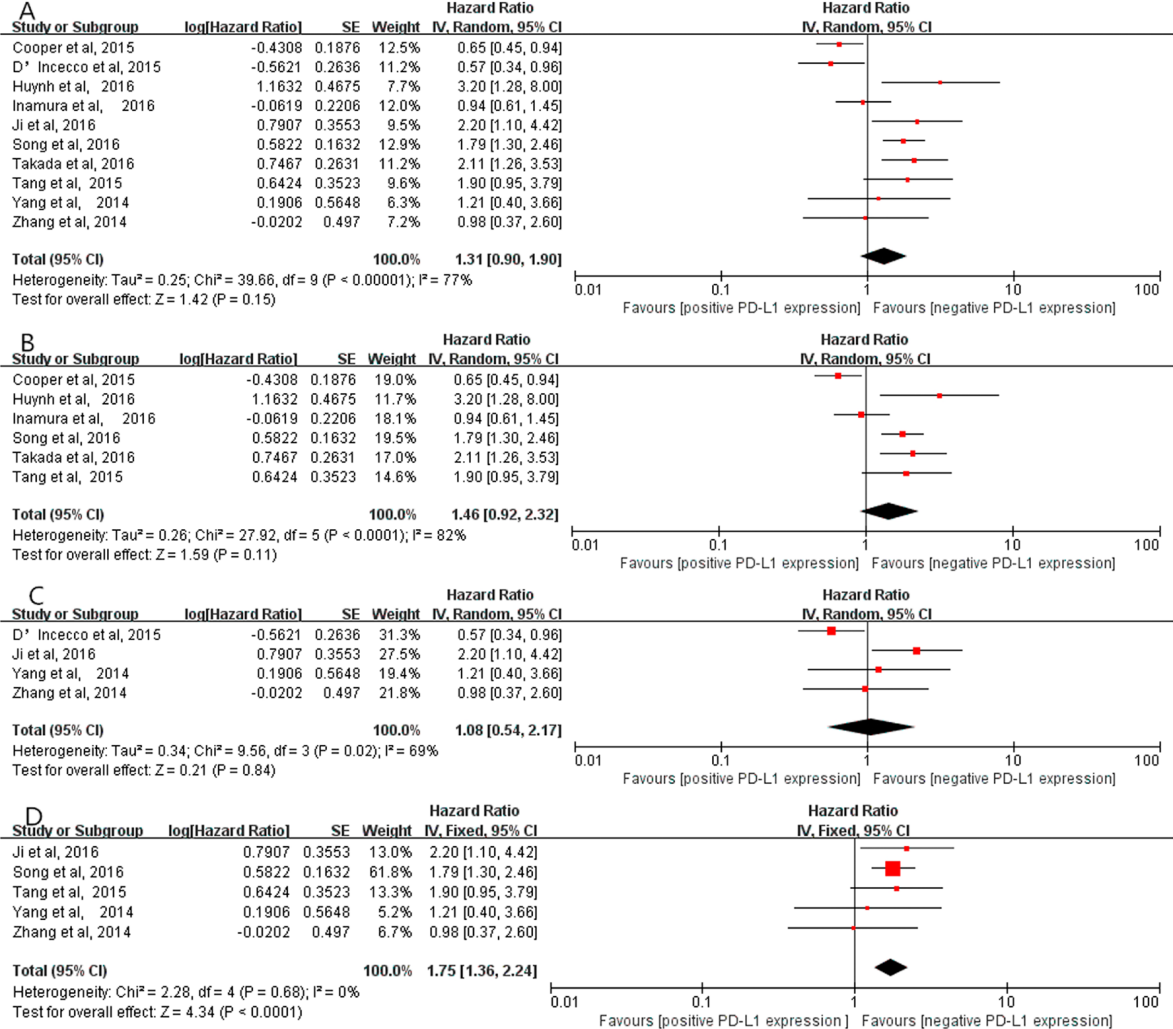
The correlation between positive PD-L1 expression and OS in overall analysis (**A**), in the studies using PD-L1 McAbs (**B**), in the studies using PD-L1 PoAbs (**C**) and in Chinese cohort studies (**D**).

Ten studies [13–22] (2708 patients) exploring the correlation between PD-L1 expression and OS were included in this study. A random-effects model was employed because of obvious heterogeneity (*I*^2^ = 77%, *P* < 0.00001). The result indicated that positive PD-L1 expression tended to be associated with poor OS, despite no statistical significance (HR=1.31, 95% CI, 0.90–1.90; *P* = 0.15, Figure 9A). Additionally, subgroup analyses on the studies using PD-L1 McAbs [13, 15, 16, 18–20] and on the studies using PD-L1 PoAbs [14, 17, 21, 22] showed that positive PD-L1 expression was not associated with OS (HR = 1.46, 95% CI, 0.92–2.32; *P* = 0.11, Figure 9B), (HR = 1.08, 95% CI, 0.54–2.17; *P* = 0.84, Figure 9C), respectively. However, in Chinese cohort studies [17, 18, 20–22], the result implied that positive PD-L1 expression was significantly related to poor OS (HR = 1.75, 95% CI, 1.36–2.24; *P* < 0.0001, Figure 9D) without obvious heterogeneity (*I*^2^ = 0%, *P* = 0.68).

### Publication bias

**Figure 10:**
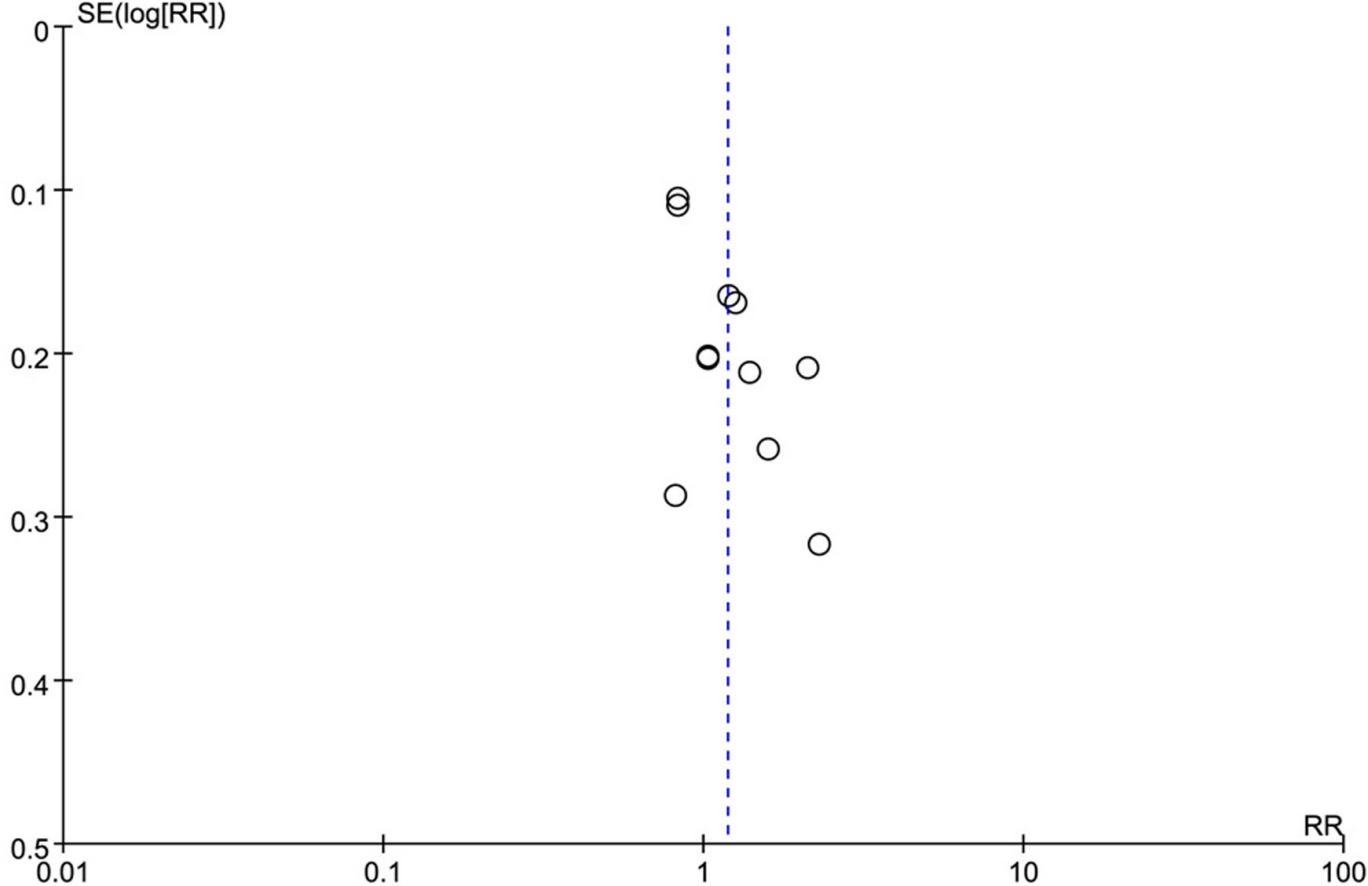
The funnel plot of the correlation between positive PD-L1 expression and gender

The funnel plot of all included studies (Figure 10) indicated that no remarkable publication bias existed in this study, suggesting that the obtained results were reliable.

## DISCUSSION

To the best of our knowledge, this study involving 11 eligible studies (3128 cases) is the first meta-analysis to focus on the correlation between positive PD-L1 expression and driver genes in NSCLC. Our study revealed that positive PD-L1 expression tended to occur more frequently in female, never smoking, ADC, EGFR mutation and ALK positive, but no significant association between them. Interestingly, there was a significant correlation between positive PD-L1 expression and KRAS status. Meanwhile, concerning the diversity of PD-L1 antibodies, further subgroup analyses based on the studies using PD-L1 McAbs or PoAbs were implemented. And the results showed that positive PD-L1 expression was significantly associated with KRAS status and EGFR status in the studies using PD-L1 McAbs, but not in the studies using PD-L1 PoAbs. Furthermore, we also conducted another subgroup analyses on Chinese cohort studies, which revealed that positive PD-L1 expression was associated with only OS, but not with other parameters.

KRAS mutation is one of the most common driver genes in NSCLC, but the aim of designing therapeutic regimen for lung ADCs harboring KRAS mutations has far proven elusive. Our study demonstrated that positive PD-L1 expression had a significant correlation with KRAS status. The result mentioned above was obtained in the studies using PD-L1 McAbs, while it did not occur in the studies using PD-L1 PoAbs and in Chinese cohort studies. One reason for the inconsistent results between overall and subgroup analyses might be the diversity of races (4 studies were from China, 1 from Japan, 2 from Austria, 1 from Italy and 1 from USA), because KRAS mutation was low expression in Chinese population [26]. The other reason might be the distinct features between McAbs and PoAbs. Generally, McAbs has a higher specificity while PoAbs possesses a higher sensitivity, especially in identifying low-abundance proteins. Recently, some oncologists also conducted studies to investigate the relationship between positive PD-L1 expression and KRAS status, finding that immune markers (including PD-L1) decreased in KRAS mutant tumors harboring STK11/LKB1 alterations but increased in KRAS mutant tumors bearing TP53 alterations [27]. Moreover, Pivarcsi et al reported that the activation of EGFR and KRAS pathways might be involved in immune response suppression in murine melanoma models [28]. On that account positive PD-L1 expression had a significant correlation with KRAS status, thereby, PD-L1 inhibitors might be a potential option in managing NSCLC harboring KRAS mutation.

With respect to the relationship between PD-L1 expression and EGFR status, some studies showed that the activation of EGFR pathway might up-regulate PD-L1 expression [29]. Our study revealed that PD-L1 expression was significantly associated with EGFR status in the studies using PD-L1 McAbs but not in the studies using PD-L1 PoAbs. The discrepancy probably be attributed to the diversity of antibodies, however, the mechanism remained to be elaborated.

Additionally, this study revealed that PD-L1 expression had no significant correlation with ALK status in both overall and subgroup analyses. However, Ota et al showed that EML4-ALK rearrangements and downstream signaling pathways could induced PD-L1 expression in NSCLC models [30]. Thus, the association between positive PD-L1 expression and ALK status still needs better designed pre-clinical trials and clinical trials to further clarify.

Furthermore, subgroup analyses demonstrated that positive PD-L1 expression was significantly associated with OS in Chinese cohort studies but not in the studies using PD-L1 McAbs or PoAbs. This differences may attribute to the diversity of races, the definition of positive/negative PD-L1 expression, the various therapeutic regimen, the duration of follow-up, the baseline characteristics as well as the potential bias of several HRs which were extracted from Kaplan-Meier curves.

Nevertheless, we also encountered some limitations: firstly, the sample size of participants was not substantial, only 11 studies containing 3128 cases, thus, more large scales studies including various races were required to further determine the correlation between PD-L1 expression and drive genes; subsequently, all eligible articles were published in English, and other languages were not included, which may be a potential bias; finally, the obvious heterogeneity among these eligible studies would affect the stability of statistical analyses to some extent, despite the fact that subgroup analyses had lowered the heterogeneity.

Accordingly, PD-L1 inhibitors probably was a potential promising option to manage advanced NSCLC harboring KRAS mutation.

## MATERIALS AND METHODS

### Data sources and search

Relevant articles were thoroughly searched from PubMed, EMBASE and Cochrane Library databases using the following terms: programmed cell death-ligand 1, PD-L1, B7-H1, lung cancer, NSCLC, EGFR, ALK, KRAS and driver genes, from their inception till September 2016. The search was performed with language limitation to English. If the same population was found in different publications, the latest or the most complete articles were included. Once the articles were identified, the references were also searched for extending the search. All potential relevant articles were scanned by two researchers (Yang HT and Chen HJ) independently via the following process: initially excluding duplication by Endnote X7 software, then selecting pertinent articles via screening titles or abstracts and eventually identifying eligible articles through scrutinizing full-texts. Disagreement was resolved by consensus or the third researcher (Xie XH).

### Study selection

The eligible articles should meet the following criteria: i) all patients had histologically or cytologically confirmed NSCLC; ii) PD-L1 expression as well as one or more driver genes (EGFR, ALK and KRAS) were available; iii) all studies investigated the correlation between positive PD-L1 expression and driver genes mutations in NSCLC and acquired sufficient data; iv) all articles were published in English. Reviews, letters, ongoing studies and insufficient data were excluded.

### Data extraction

Two researchers (Yang HT and Chen HJ) extracted the following data independently from all eligible studies: the first author's name, year of publication, country, gender, smoking status, histological type, PD-L1 expression (positive/negative), driver genes (EGFR, ALK and KRAS status) and OS. When the extracted data existed discrepancy, consultation was held to settle the problem.

### Statistical analysis

The pooled data were calculated via Review manager 5.3 software. Analyses were conducted according to gender, smoking status, histological type, driver genes (EGFR, ALK and KRAS status) and OS. The effective value, RR with 95% confidence intervals (95% CIs), was employed to evaluate the correlation between positive PD-L1 expression and driver gene mutations, clinical characteristics in NSCLC. Hazard ratios (HRs) with 95% CIs were extracted from the articles or calculated from survival curves according to Zhou et al method if these data were unavailable [31]. HR>1 indicated that positive PD-L1 expression had a poor prognosis in NSCLC. Heterogeneity were measured by Chi-square and I-square tests [32]. If *P <* 0.1 and I^2^ > 50%, indicating significant heterogeneity, a random effects model was utilized, otherwise, a fixed-effect model was employed [33]. With regard to the diversity of PD-L1 antibodies and nations, subgroup analyses were conducted on Chinese cohort studies, the studies using PD-L1 McAbs or PD-L1 PoAbs, respectively. Publication bias was assessed by funnel plot.

## Abbreviations

EGFR: epidermal growth factor receptor
KRAS: Kirsten rat sarcoma viral oncogene homolog
ALK: anaplastic lymphoma kinase
NSCLC: non-small cell lung cancer
SCLC: small cell lung cancer
PD-L1: programmed death-ligand 1
PD-1: programmed death 1
OS: overall survival
PoAbs: polyclonal antibodies
McAbs: monoclonal antibodies
95% CI: 95% confidence interval
HR: hazard ratio
M/F: male/female
ADC: adenocarcinoma
non-ADC: non-adenocarcinoma
N/P: negative/positive
NA: unavailable
